# Halochromic coordination polymers based on a triarylmethane dye for reversible detection of acids†
†Electronic supplementary information (ESI) available: Synthesis and analytical methods, crystallographic information, IR spectra, TGA plots and additional figures and photographs. CCDC 1504745 and 1504746. For ESI and crystallographic data in CIF or other electronic format see DOI: 10.1039/c6dt03969c



**DOI:** 10.1039/c6dt03969c

**Published:** 2016-12-05

**Authors:** Marina S. Zavakhina, Irina V. Yushina, Denis G. Samsonenko, Danil N. Dybtsev, Vladimir P. Fedin, Stephen P. Argent, Alexander J. Blake, Martin Schröder

**Affiliations:** a Nikolaev Institute of Inorganic Chemistry , Russian Academy of Sciences , Siberian Branch , Acad. Lavrentiev Ave. , 3 , Novosibirsk , 630090 , Russian Federation . Email: dan@niic.nsc.ru ; Fax: +7 (383) 330 9489; b Novosibirsk State University , Pirogov str. , 2 , Novosibirsk , 630090 , Russian Federation; c School of Chemistry , University of Nottingham , NG7 2RD Nottingham , UK; d School of Chemistry , University of Manchester , M13 9PL Manchester , UK

## Abstract

Chromeazurol B (Na_2_HL) is a pH-sensitive (halochromic) dye based on a hydroxytriarylmethane core and two carboxylate functional groups, which makes it suitable for the synthesis of coordination polymers. Two new coordination polymers [NaZn_4_(H_2_O)_3_(L)_3_]·3THF·3H_2_O (**1**) and [Zn_3_(H_2_O)_3_(μ_2_-OH_2_)(μ_3_-OH)(HL)_2_(H_2_L)]·2THF·3H_2_O (**2**) incorporating Chromeazurol B linkers have been prepared and characterised. The structure of **1** comprises pentanuclear heterometallic {Zn_4_Na} nodes linked by six L^3–^ anions to give a layered structure with a honeycomb topology. **2** crystallizes as a double-chain ribbon (ladder) structure with two types of metal node: a mononuclear Zn(ii) cation and tetranuclear {Zn(ii)}_4_ cluster. Chromeazurol B anions link each tetranuclear cluster to four individual Zn(ii) cations and each Zn(ii) cation with four tetranuclear clusters. Both compounds show pH-sensitivity in water solution which can be observed visually, giving the first example of a halochromic coordination polymer. The halochromic properties of **1** towards HCl vapors were systematically investigated. As-synthesized violet-grey **1** reversibly changes color from orange to pink in the presence of vapors of 2 M and 7 M HCl, respectively. The coordination of the Chromeazurol B anion at each color stage was examined by diffuse reflectance spectroscopy and FT-IR measurements. The remarkable stability of **1** to acid and the observed reversible and reproducible color changes provide a new design for multifunctional sensor materials.

## Abstract 2

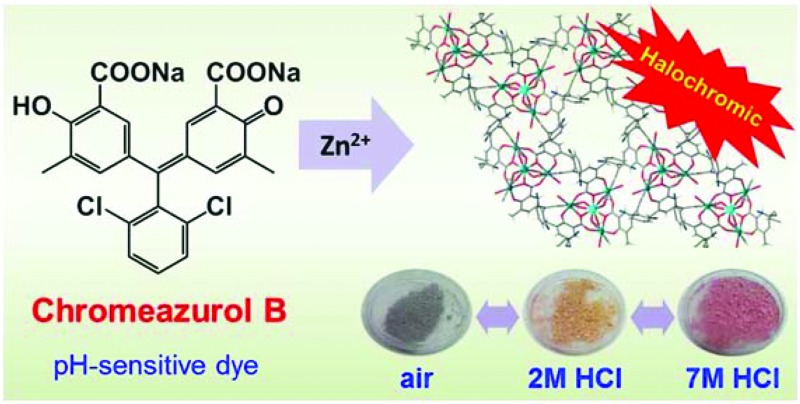
A pH-sensitive coordination polymer reversibly changes its color in air or acidic atmosphere.

## Introduction

Metal–organic frameworks (MOFs) are hybrid materials assembled from metal ions or clusters and organic linkers,^1^ and their adaptable modular design results in various tailored properties for the resultant porous materials^2^ including gas storage and separation properties.^3^ The use of porous MOFs as sensors is another important direction and is receiving increasing attention.^4^ Compared to molecular sensors, porous MOFs have a number of advantages, such as potentially higher stability, and, most importantly, the microporous structure with extended surface area can ensure effective adsorption of analyte molecules from gas or liquid media into the pores, thus potentially improving detection limits. Also, by tuning the pore geometry and functionality, the crystalline MOF sensor can be adapted for the detection of a specific molecule within a set of complex mixtures. There are many interesting examples of MOFs used for the detection of explosives,^5^ hazardous organic substances,^6^ and metal cations.^7^ These properties are typically based on magnetic,^8^ electrochemical/electromechanical^9^ or luminescent^10^ response of the porous framework upon inclusion of the particular guest molecule. Although luminescence sensors possess good detection levels, an external UV light source is usually required for visualization. In contrast, colorimetric sensors featuring a change of color of the material by an external stimulus can potentially be monitored visually and are therefore simpler to use. To date, however, there are few reports on colorimetric sensing by MOFs in which different porphyrinic,^11^ diimide,^12^ or viologen-substituted ligands^13^ have been employed. In another example based on Co(ii), a color change occurs when the metal cation changes coordination environment from octahedral to tetrahedral.^14^

**Fig. 1:**
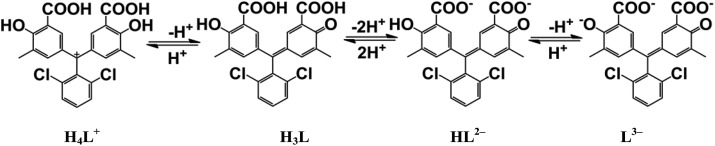
View of different forms of Chromeazurol B in solution.

Halochromic compounds (pH indicators) are among the most commonly used sensors in chemistry. Such molecules often possess a system of conjugated bonds which are altered upon protonation or deprotonation. Hydroxytriarylmethane-based pH indicators are classic examples of such systems.^15^ These molecules, if incorporated into a coordination polymer, *e.g.*, within the linkers of a MOF, could function as halochromic detectors within the resultant material. Strikingly, this idea, despite being rather simple and straightforward, has not to our knowledge been explored previously. Chromeazurol B (also known as Chrome Pure Blue BX, Eriochrome Azurol B, Mordant Blue 1, herein denoted as Na_2_HL) is a well-known pH-sensitive compound based on hydroxytriarylmethane core.^16^ Depending on its protonation level, it exists in one of four different forms, each having a distinct color, thus representing a one-component universal pH sensor (Fig. 1). It also bears two carboxylate groups in its structure suggesting its potential use as a bridging ligand within a MOF architecture. Although a few structurally characterized molecular coordination compounds based on hydroxyarylmethane ligands have been reported, their pH dependent halochromic behavior has not been investigated.^17^

In this work we present two new coordination polymers [NaZn_4_(H_2_O)_3_(L)_3_]·3THF·3H_2_O (**1**) and [Zn_3_(H_2_O)_3_(μ_2_-OH_2_)(μ_3_-OH)(HL)_2_(H_2_L)]·2THF·3H_2_O (**2**) based on the Chromeazurol B dye as a bridging linker, and report their optical properties for **1** as a function of pH. These compounds are the first examples of MOF materials that show halochromic behavior in aqueous solution as well as with acidic vapors. The observation of reversible color changes and the structural stability of **1** at low pH demonstrate its pH sensing capabilities and opens new perspectives for MOF materials in important sensing applications.

## Results and discussion

### Protonation levels of Chromeazurol B (Na_2_HL)

**Fig. 2:**
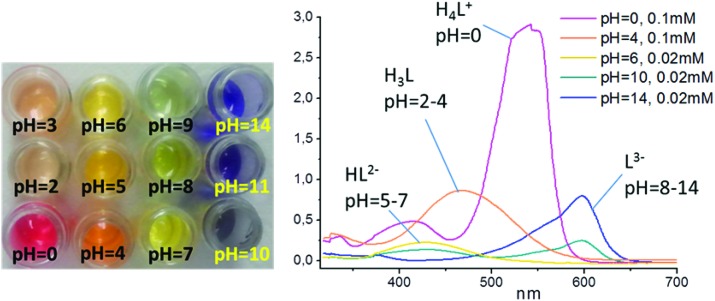
Photographs and UV-vis spectra of Chromeazurol B in aqueous solutions of different pH.

The dependence of Chromeazurol B color with pH was examined by spectrophotometry in solution for pH = 0–14. Four absorbance bands (550 nm, 480 nm, 430 nm, 600 nm) in the visible spectrum can be assigned to 4 different forms of the dye (Fig. 2).^18^ At pH = 0 the pink-red protonated form H_4_L^+^ is formed (*ε*(550 nm) = 2.8 × 10^4^ M^–1^ cm^–1^), while at pH = 2–4 the less intense red-orange form H_3_L (*ε*(H_3_L, 480 nm) = 8.6 × 10^3^ M^–1^ cm^–1^) is formed and at pH >4 a yellow-orange form HL^2–^ (*ε*(430 nm) = 1.1 × 10^4^ M^–1^ cm^–1^) exists. Finally, the fully deprotonated blue form of Chromeazurol B L^3–^ (*ε*(600 nm) = 3.9 × 10^4^ M^–1^ cm^–1^) is formed in highly basic solutions.^15,16^

### Synthesis

Compounds [NaZn_4_(H_2_O)_3_(L)_3_]·3THF·3H_2_O (**1**) and [Zn_3_(H_2_O)_3_(μ_2_-OH_2_)(μ_3_-OH)(HL)_2_(H_2_L)]·2THF·3H_2_O (**2**) were obtained by reaction of Chromeazurol B Na_2_(HL) with Zn(OAc)_2_ in water/THF solution. The solvent composition is critical, because compound **1** is obtained from a 2 : 1 mixture of water : THF, while **2** can be isolated from the more polar 4 : 1 water : THF solvent mixture. Both crystalline products are deeply colored and appear almost black. Crystals of the compound **1** adopt a rhombohedral shape with a dark golden hue, while **2** forms octahedral crystals with a greenish luster, red in transmitted light (Fig. S1†).

### Structures

#### [NaZn_4_(H_2_O)_3_(L)_3_]·3H_2_O·3THF (**1**)

**Fig. 3:**
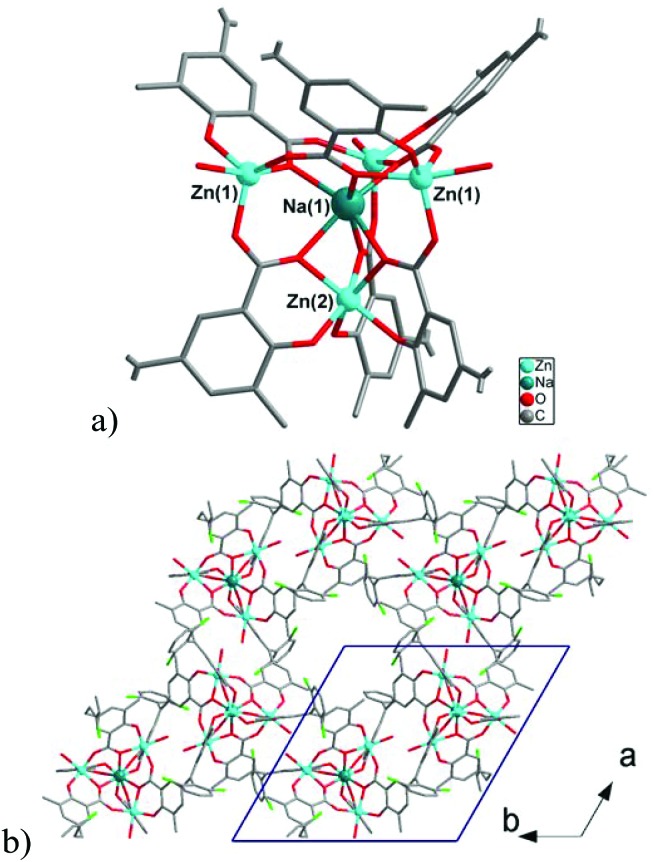
(a) View of {NaZn_4_} cluster in **1**. (b) The structure of the layer in **1**. Hydrogen atoms are omitted for clarity.

Compound **1** shows a layered structure constructed around a pentanuclear {Zn_4_Na} cluster (Fig. 3a) comprising of two types of Zn(ii) cations [three Zn(1) and one Zn(2)] and one Na(1) cation. The Zn(1) cation shows a distorted trigonal bipyramidal coordination environment formed by an aqua ligand and four carboxylate O-donors of three ligands L^3–^. The Zn(2) center has a distorted octahedral geometry formed by six O-donors from three carboxylate and three phenolate moieties of L^3–^. All Zn–O distances are in range 1.9618(18)–2.2216(19) Å, and Na(1) has distorted octahedral coordination environment formed by six carboxylate oxygen atoms from six ligands L^3–^ (Na–O = 2.2573(19)–2.334(2) Å). Each pentanuclear {Zn_4_Na} building unit is bound to six Chromeazurol linkers, each of which bridges two Zn_4_Na units to form layers (Fig. 3b) of honeycomb topology (Fig. S7†). The X-ray diffraction analysis also reveals three THF guest molecules per formula unit and some residual electron density assigned to a disordered water molecules in the void space in **1** (*ca*. 3H_2_O molecules per formula unit according to PLATON/SQUEEZE^19^). Elemental analysis data confirm the composition of the compound. Thermogravimetric analysis (TGA) of **1** indicates an ill-defined two-step weight loss up to 250 °C (18%), attributed to both guest and coordinated solvent molecules (calc. 16.3%), followed by the framework decomposition.

#### [Zn_3_(H_2_O)_3_(μ_2_-OH_2_)(μ_3_-OH)(HL)_2_(H_2_L)]·2THF·3H_2_O (**2**)

**Fig. 4:**
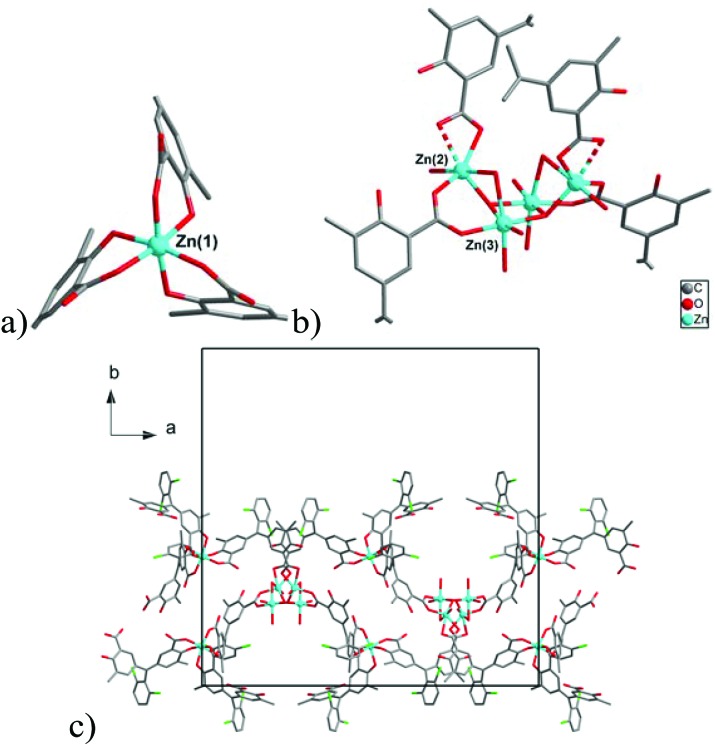
Views of (a) the coordination environment of the Zn(1) cation in **2**, (b) the structure of tetranuclear cluster in **2**, and (c) the structure of the polymeric chain in **2**. Hydrogen atoms are omitted for clarity.

Compound **2** has a chain structure with the asymmetric unit containing three Zn(ii) cations and three Chromeazurol B ligands, one as H_2_L^–^ and two as HL^2–^. Zn(1) has octahedral coordination formed by six O-donors (three of carboxylate and three of phenolate moieties) of three organic ligands (Fig. 4a). Two Zn(2) and two Zn(3) centers form a tetranuclear building unit {Zn_2_(H_2_O)_3_(μ-OH_2_)(μ_3_-OH)(OOCR)_2_}_2_ (Fig. 4b), whereas the Zn(1) centers form part of mononuclear units. Zn(2) has a distorted octahedral coordination formed by three carboxylate oxygen atoms of two organic ligands, two aqua-ligands (one terminal and one bridged), and a bridging μ_3_-OH group. Zn(3) has slightly distorted octahedral coordination environment formed by a carboxylate oxygen, three aqua-ligands (two terminal and one bridged), and two μ_3_-OH groups. Zn(2) connects to Zn(3) *via* bridged carboxylate group of the organic ligand, a μ_2_-OH_2_, and a μ_3_-OH group. Zn(3) connects to another Zn(3) *via* two μ_3_-OH ligands with each μ_3_-OH ligand connecting one Zn(2) and two Zn(3) cations (Fig. 3b). Each tetranuclear unit connects with four Zn(1) cations *via* four bridging HL^2–^ ligands, while each Zn(1) cation connects with two tetranuclear units *via* two bridging HL^2–^ ligands. The H_2_L^–^ moiety is coordinated to Zn(1) as a terminal ligand. Thus, Zn(1) centers can be described as two–connected nodes, while the tetranuclear units act as four-connected nodes. Interconnection of these coordination nodes forms form polymeric chains (Fig. 4c) with the unit cell containing 96 molecules of Chromeazurol B and 96 Zn atoms (32 mononuclear and 16 tetranuclear units) to give an overall formula [Zn_3_(H_2_O)_3_(μ_2_-OH_2_)(μ_3_-OH)(HL)_2_(H_2_L)]. The interstitial space is occupied by solvent THF and water molecules, and the tentative guest composition (two THF and three H_2_O molecules per formula unit) was established from elemental analysis data and thermal gravimetric analysis (TGA). TGA shows a multistep weight loss curve with the first step (4% loss at 100 °C) attributed to 1 THF molecule (calc. 3.9%) and the second step (12%, 150 °C) to the removal of the remaining guest molecules (calc. 10.7%), followed by a decomposition of the coordination polymer above 170 °C.

### Sensing properties

**Fig. 5:**
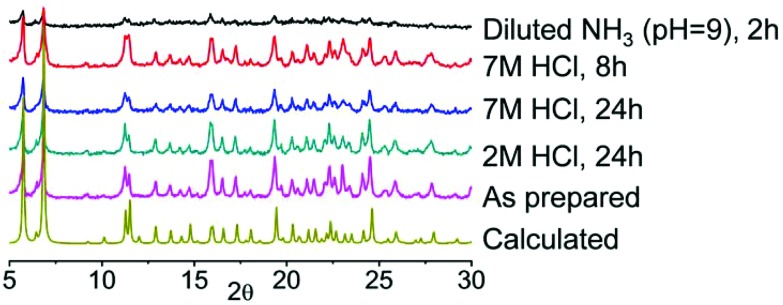
PXRD patterns for **1** (calculated) and for **1** under different vapors of 2 M HCl, 7 M HCl, NH_3_ solution at pH 9. Exposure time is 24 h for 2 M acid vapors, 24 h and 8 h for 7 M HCl vapors, and 2 h for NH_3_.

Successful synthesis of new coordination compounds incorporating Chromeazurol B prompted us to investigate the halochromic behavior of these materials. Preliminary experiments with thin layer samples of **1** and **2** in both water and dilute HCl solution (pH = 2) indicate that they have similar behavior. The color of crystals of both compounds changes reversibly from violet to orange by varying the pH of the media from neutral to acidic (Fig. S9†). Because of their similarity in pH behavior and the better crystallinity and quality for **1**, we investigated the detailed halochromic properties of **1** only. The stability of **1** under a broad range of acidic and basic conditions was examined by powder X-ray diffraction analysis (Fig. 5). When exposed to vapors^20^ of an aqueous solution of 2 M HCl (partial pressure of HCl gas *ca*. 10^–3^ mmHg), **1** retains its crystallinity for at least 1 day. Moreover, under vapors of 7 M HCl (partial pressure of HCl gas *ca*. 0.64 mmHg) the crystals of **1** remain stable for at least 8 h and even after 1 day of exposure the quality of the diffraction pattern remains good. However, under vapors of aqueous NH_3_ solution at pH 9 **1** decomposes rapidly and thus were only able to conduct sensing experiments under acidic atmospheres only.

**Fig. 6:**
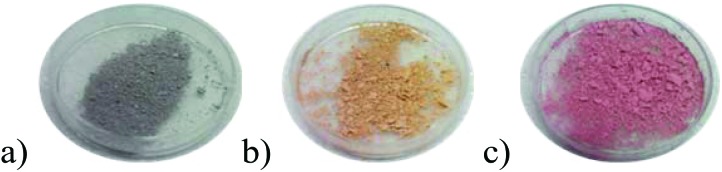
Views of **1**@BaSO_4_ (5% in BaSO_4_): (a) in air, (b) under 2 M HCl vapor and (c) under 7 M HCl vapor.

The halochromic properties of **1** were investigated by a number of methods. Because of the intense absorbance of **1**, the crystals were ground and mixed with BaSO_4_ (5% of **1** in BaSO_4_ by weight). A thin layer of the corresponding powder mixture was exposed to vapors of solutions of HCl of different concentrations (2 M to 7 M). The violet-gray color of the as-synthesized **1**@BaSO_4_ turned orange in 2 M and pink in 7 M HCl (Fig. 6a–c). When exposed to HCl vapors of intermediate concentration between 4 M and 6 M HCl the sample exhibited intermediate colors (Fig. S10†). In 7 M HCl the color of the sample changed over a few minutes, while in 2 M the transition takes few hours (Fig. S11†). It is important to note that all changes were completely reversible since pink and orange powders turned back to violet-gray after aeration, though the complete release of acid is a slower process than its uptake.

**Fig. 7:**
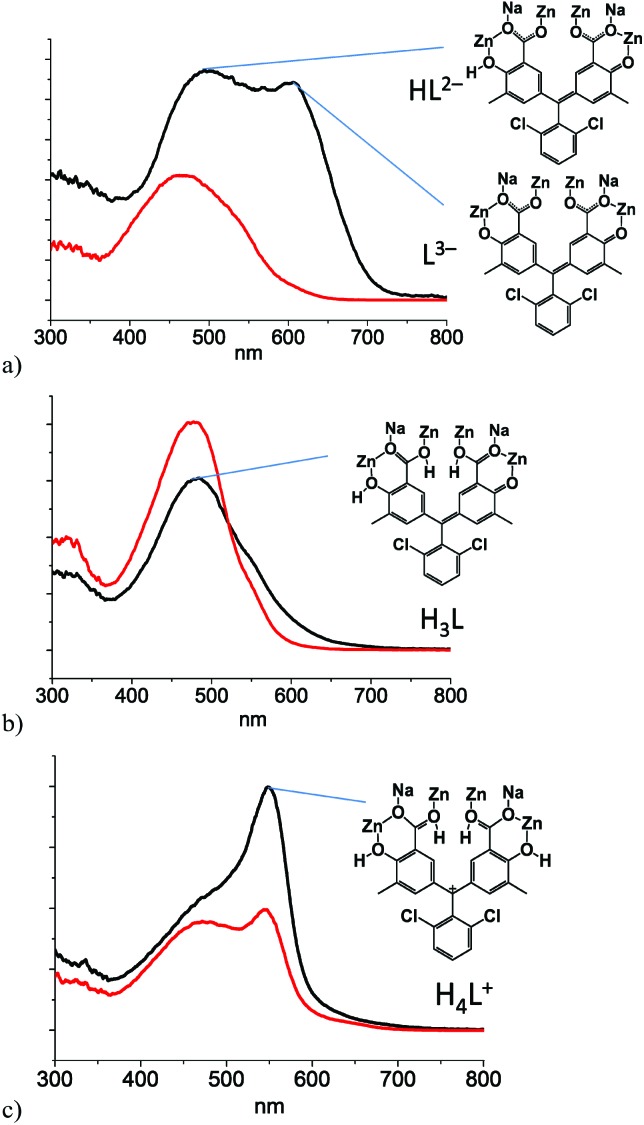
Absorbance spectra and forms of the organic anion in **1**: (a) under air, (b) under 2 M acid vapor and (c) under 7 M acid vapor. Black lines are for **1** and the red lines for Na_2_HL.

Diffuse reflectance spectroscopy (DRS) measurements clearly supported the visual observations as the spectra of the pelleted samples of **1**@BaSO_4_ exposed to various media revealed remarkable changes depending on the acidity of the atmosphere (Fig. 7). For comparison we also carried out the DRS measurements of pellets of Na_2_HL@BaSO_4_, treated similarly to **1**@BaSO_4_. The positions of the reflectance peaks for solid samples Na_2_HL@BaSO_4_ are similar to the adsorption bands in the UV-VIS spectra of Chromeazurol B in aqueous solutions at similar acidity. Also, the observed DRS peaks of **1**@BaSO_4_ closely match those for the free ligand Na_2_HL@BaSO_4_. Therefore, coordination of Zn(ii) to donor groups of the Chromeazurol B ligand does not seem to change the ability of the latter to accept the protons from the acidic environment. In other words, particular protonated forms of the pH-sensitive ligand exist in the coordination polymer **1** as in free Chromeazurol B under comparable conditions.

The DRS data for as prepared Na_2_HL@BaSO_4_ show only the component HL^2–^ at *ca*. 460 nm (ref. 17) while the spectrum of **1**@BaSO_4_ consists of two absorption bands at *ca*. 480 and 620 nm (Fig. 7a), assigned to the two anionic forms L^3–^ and HL^2–^, respectively, although only L^3–^ is assumed in the crystal structure of **1** according to the requirement of charge-neutrality. The monoprotonated species HL^2–^ probably exists in the crystalline compound as a result of the partial protonation of basic phenolate group by a solvent water and/or airborne moisture, which cannot be identified from the single-crystal X-ray diffraction data. During exposure of Na_2_HL@BaSO_4_ to 2 M HCl vapor the band at 460 nm undergoes a bathochromic shift to 470 nm, supporting the formation of the triply-protonated charge neutral form H_3_L (Fig. 7b). There is also a similar absorption band in the reflectance spectrum of **1**@BaSO_4_ under 2 M HCl vapors so we can assume full protonation of the bound Chromeazurol B ligand in **1** under these conditions. Finally, when exposed to vapors of 7 M HCl the sample Na_2_HL@BaSO_4_ turned pink as does free Chromeazurol B in highly acidic solutions. This color and the major absorption band at *ca*. 550 nm are characteristic of the fully protonated H_4_L^+^ species (Fig. 7c). The diffuse reflectance spectrum of **1**@BaSO_4_ exposed to 7 M HCl vapors has a similar pink color and, much like the free Chromeazurol B sample, displays the second component at *ca*. 475 nm, along with a major band at 550 nm. This suggests the simultaneous presence of H_3_L as well as H_4_L^+^ species in the samples. It should be noted that the DRS measurements and all required manipulations with the powder samples were carried out under air which may result in a partial reduction of the protonation level of the samples due to the release of volatile HCl. This explains the presence of the H_3_L component in the spectra of both samples Chromeazurol B and **1** exposed to the 7 M HCl vapors with the highest acid content. The obtained DRS data indicate that within the studied range of conditions the Chromeazurol B ligand undergoes the transition between three forms (HL^2–^, H_3_L and H_4_L^+^) while framework **1** between four forms (L^3–^, HL^2–^, H_3_L and H_4_L^+^), which makes the visual changes more characteristic and demonstrates the potential advantage of halochromic MOFs over molecular pH sensors.

**Fig. 8:**
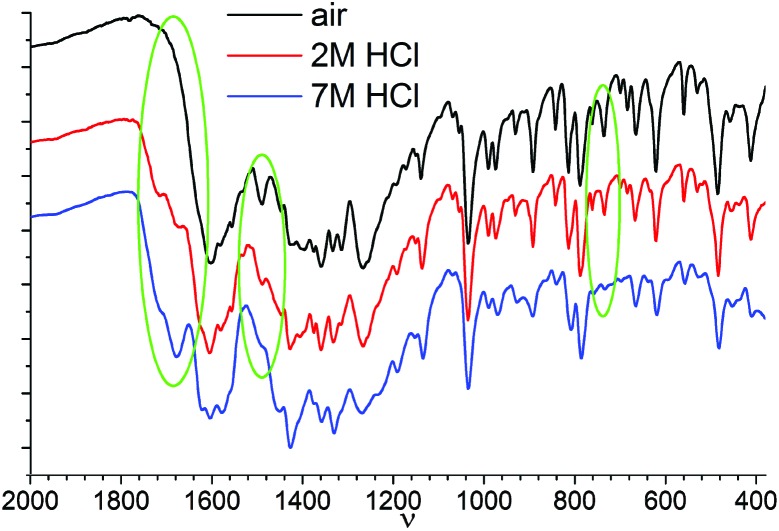
IR spectra of **1** exposed in the air (black), in 2 M HCl vapors (red) and in 7 M HCl vapors (blue).

Independent confirmation of the suggested protonated forms of Chromeazurol B ligand in crystalline **1** under different atmosphere (air, 2 M or 7 M HCl vapors) could be obtained by IR spectroscopy (Fig. 8 and S13†). Notable changes are observed for peaks at 735 and 1490 cm^–1^ assigned to vibrations of the Zn(ii) bound carboxylate anion COO^–^, and for the distinct peak at 1620 cm^–1^ assigned to the *ν*_C=O_ stretching vibration of the protonated carboxylate group bound to Zn(ii).^21^ Along with the increase in acidity of the media and hence the protonation level of **1**, the peaks for Zn(ii)-bound COO^–^ gradually disappear while the corresponding peaks for Zn(ii)-bound COOH emerge. Note, that the IR spectroscopic data support the structural models displayed in Fig. 7.

We also estimated the level of protonation of **1** under vapors of 2 M or 7 M HCl after 1 h. The corresponding microcrystalline samples were dispersed in 3 ml of a distilled H_2_O and the acidities of the resultant solutions measured using a pH sensitive electrode. The amount of the acidic protons (degree of protonation), calculated from the pH values were found to be 2.8 and 3.9 per ligand L for the samples saturated at 2 M HCl and 7 M HCl, respectively (ESI Table S4†). These match very well to the theoretical protonation levels of the Chromeazurol B ligand (*i.e.*, 3 and 4, respectively), proposed for the corresponding samples (Fig. 7b and c).

**Fig. 9:**
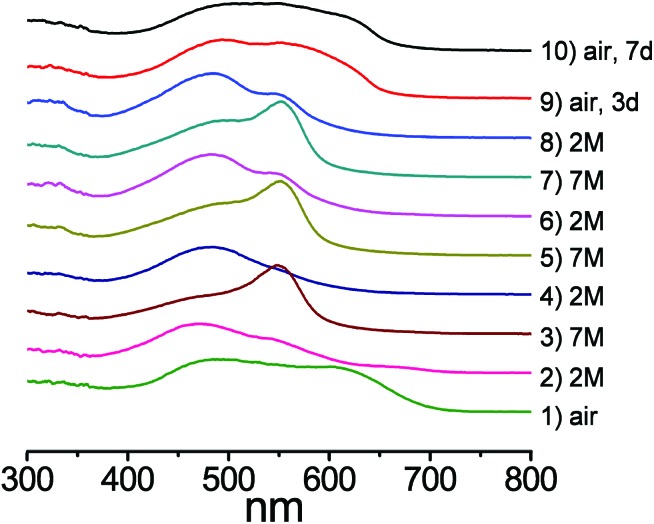
From bottom to top: DRS data of a series of consecutive experiments of exposure for **1**@BaSO_4_ to vapors from HCl of different molarity. Exposure time for each experiment is 1 h, unless otherwise stated.

The fully reversible nature of the halochromic properties of **1** was demonstrated when **1**@BaSO_4_ sample was alternately treated with 2 M HCl and 7 M HCl vapors several times (Fig. 9). The DRS data indicate reversible changes of the spectra supporting the corresponding visual changes of the sample within a few minutes or half an hour, depending on the HCl concentration (Fig. S14†). As noted above, the residual peaks at 550 nm in the compound exposed to 2 M HCl apparently result from the incomplete deprotonation of the sample. Note, however, that after these repeating experiments in the acidic atmosphere conditions the sample could be completely regenerated to its original color and spectra by aeration for few days.

## Conclusions

The present work describes the synthesis and structural characterization of two coordination compounds based on Chromeazurol B dye. The products incorporate the pH-sensitive properties of the ligand resulting in coordination polymers with hitherto unprecedented halochromic properties. Various spectroscopic techniques provide the insights into the protonation levels of this pH-sensitive system. The marked stability of **1** and **2** from pH-neutral aqueous solutions to highly acidic HCl fumes, and the reversible and reproducible character of the corresponding color changes provide new opportunities and directions for MOFs in the design and development of multifunctional sensor materials.
