# Supplementary material for: Halochromic coordination polymers based on a triarylmethane dye for reversible detection of acids
Source: Dalton Trans. 2016 Dec 5;46(2):465–70. doi: 10.1039/c6dt03969c (PMC5436038; doi:10.1039/c6dt03969c)
Supplement: Supplementary file 1 [file DT-046-C6DT03969C-s001.pdf]

Electronic Supplementary Material (ESI) for Dalton Transactions.
This journal is © The Royal Society of Chemistry 2016

ELECTRONIC SUPPORTING INFORMATION

Halochromic Coordination Polymers Based on a Triarylmethane Dye for Reversible Detection of Acids

Marina S. Zavakhina,[a,b] Irina V. Yushina,[a] Denis G. Samsonenko,[a,b] Danil N. Dybtsev,[a,b] Vladimir P. Fedin,[a,b]* Stephen P. Argent,[c] Alexander J. Blake,[c] and Martin Schröder[a,d]

[a] *Nikolaev Institute of Inorganic Chemistry, Russian Academy of Sciences, Siberian Branch, Acad. Lavrentiev Ave., 3, Novosibirsk, 630090, Russian Federation.*

Fax: +7 (383) 330 9489. E-mail: cluster@niic.nsc.ru

[b] *Novosibirsk State University, Pirogov str., 2, Novosibirsk, 630090, Russian Federation.*

[c] *School of Chemistry, University of Nottingham, NG7 2RD Nottingham, UK*

[d] *School of Chemistry, University of Manchester, M13 9PL Manchester, UK*

Contents

1. Experimental S2
2. X-ray Crystallography S4
3. Investigation of halochromism S11
4. References S14

1. Experimental

Materials and Methods. Chromeazurol B was purchased from TCI and used as delivered. Spectrophotometric experiments were carried out on ThermoScientific Helios Hamma Spectrophotometer with 1cm acrylic cuvettes. FT-IR spectra of samples (KBr pellets) were recorded on a Scimitar FTS 2000 instrument. Elemental analysis was carried out on a EURO EA 3000 instrument (EuroVector). Thermogravimetric analysis was carried out on a TG 209 F1 thermobalance (Netzsch); the samples were heated under N_2/O_2 (4/1) with a heating rate of 10 deg·min^{-1}. PXRD patterns were measured on a Shimadzu XRD 7000S automated powder diffractometer (Cu-Kα radiation, λ = 1.54056 Å).

Synthesis of [NaZn$_4$(H$_2$O)$_3$(L)$_3$]·3THF·3H$_2$O. Zn(OAc)$_2$·2H$_2$O (11mg, 0.05 mmol) and Na$_2$HL (25 mg, 0.05 mmol) were dissolved in water/THF mixture (5 ml, 2:1). The mixture was heated at sealed glass tube at 100°C for 3 days after which black rhombohedral crystals (Fig. S1a) with golden sheen were formed. The glass tube was left at room temperature for 4 days during which crystals continued to precipitate. The resulting product was collected, washed with aqueous HCl solution (pH = 3) and dried at the air. Yield: 35 mg (47% based on metal). Elemental analysis for [NaZn$_4$(H$_2$O)$_3$(C$_{23}$H$_{13}$C$_{l2}$O$_6$)$_3$]·3C$_4$H$_8$O·3H$_2$O: Calculated: C 49.19; H 3.82; found: C 49.2; H 3.7. IR (KBr): ν = 411 w, 485 s, 560 w , 621 m, 665 w, 735 w, 788 m, 814 m, 842 w, 892 w, 931 w, 975 m, 990 m, 1035 s, 1139 m, 1266 s, 1314 s, 1334 s, 1360 s, 1395 s, 1490 m,1556 m, 1582 m, 1605 s, 2873 m, 2960 m, 3425 m, 3533 m. It is also possible to obtain crystals **1** not only from THF/water mixture, but also from water/dioxane solutions. In this case the resulting product is contaminated with red needle crystals which could not be separated and gave no diffraction. To obtain pure **1**, we used THF/water solutions only.

Synthesis of [Zn$_3$(H$_2$O)$_3$(μ$_2$-OH$_2$)(μ$_3$-OH)(HL)$_2$(H$_2$L)]·2THF·3H$_2$O Zn(OAc)$_2$·2H$_2$O (11mg, 0.05 mmol) and Na$_2$HL (25 mg, 0.05 mmol) was suspended in water/THF mixture (5 ml, 4:1). The mixture was heated in a sealed glass tube at 100°C for 3 days. After cooling and standing for 2 days black octahedral crystals (with green sheen; red in transmitted light) were collected (Fig. S1b), washed with HCl solution (pH 3) and dried in air. Yield: 10 mg (31%). Elemental analysis for [Zn$_3$(H$_2$O)$_3$(μ$_2$-OH$_2$)(μ$_3$-OH)(HL)$_2$(H$_2$L)]·2C$_4$H$_8$O·3H$_2$O: Calculated: C 50.00; H 4.00; found: C 50.1; H 4.1. IR (KBr): ν = 485 s, 601 w , 655 m, 672 m, 734 w, 785 s, 815 m, 894 m, 866 m, 1034 s, 1135 m, 1206 w, 1278 s, 1325 s, 1426 s, 1468 s, 1498 m, 1594 s,1629 s, 2871 m, 2918 m, 2947 m, 2973 m, 3367 m.

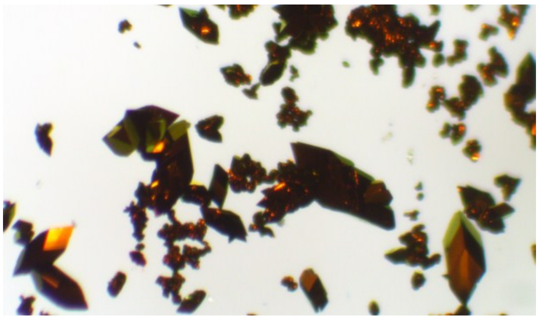

a

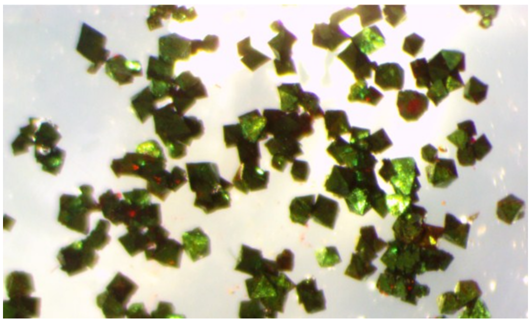

b

Figure S1. Views of (a) crystals of $[NaZn_4(H_2O)_3(L)_3]\cdot3THF\cdot3H_2O$, **1** and (b) crystals of $[Zn_3(H_2O)_3(\mu_2\text{-}OH_2)(\mu_3\text{-}OH)(HL)_2(H_2L)]\cdot2THF\cdot3H_2O$, **2**.

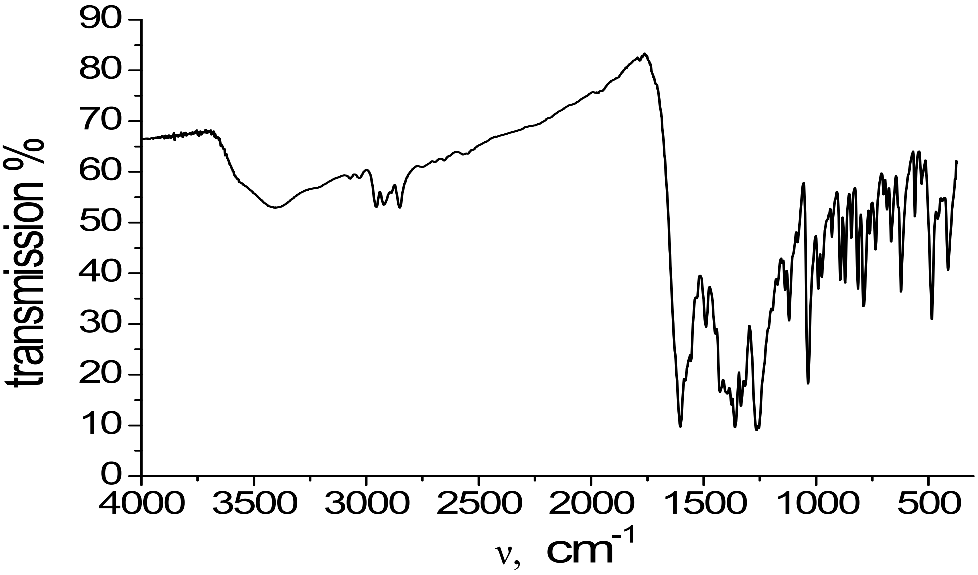

Figure S2. IR spectrum of **1**.

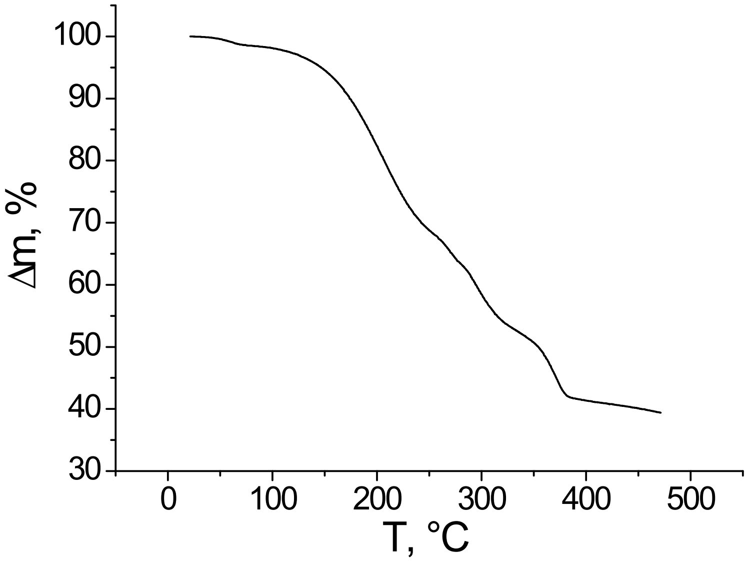

Figure S3. Thermogravimetric analysis of **1**.

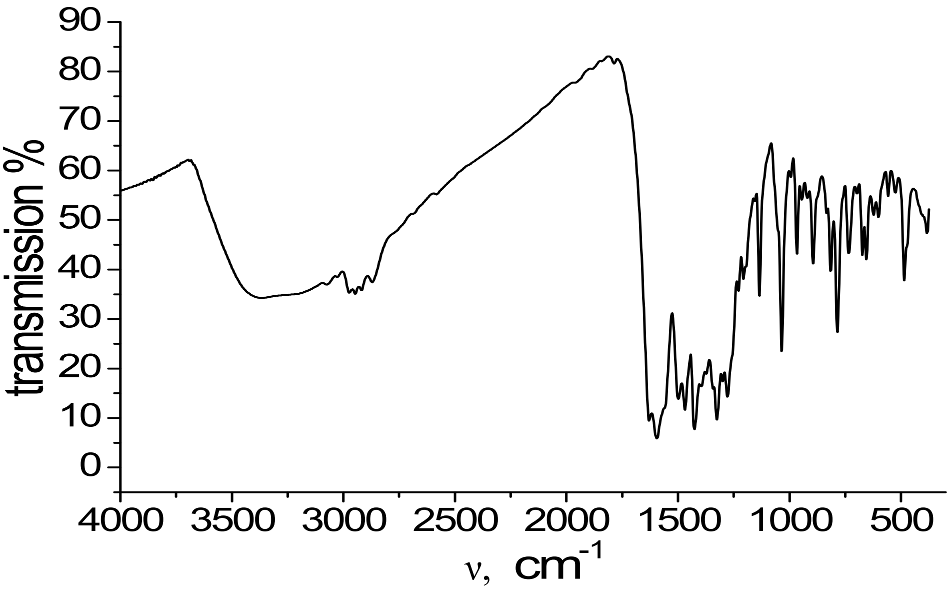

Figure S4. IR spectrum of **2**.

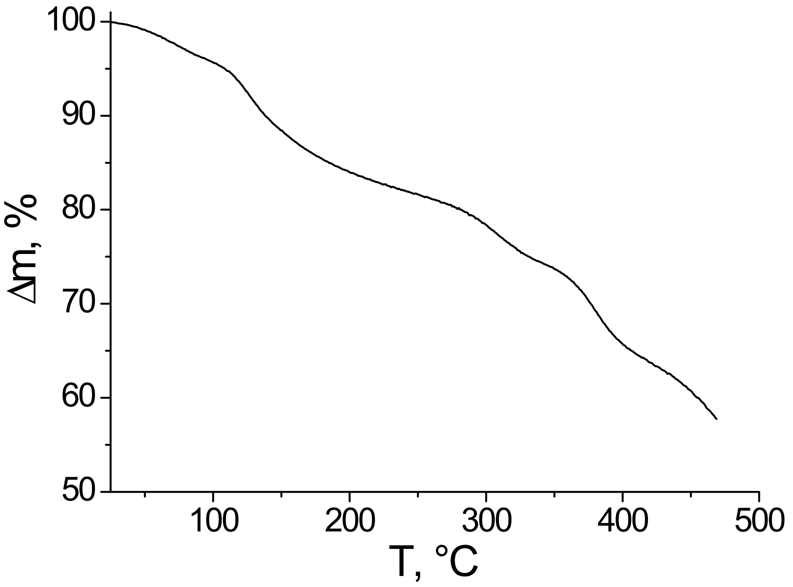

Figure S5. Thermogravimetric analysis of **2**.

2. X-Ray Crystallography

Diffraction data for a single crystal of **1** were obtained at 130 K on an automated Agilent Xcalibur diffractometer equipped with a two-dimensional AtlasS2 detector (graphite monochromator, λ(MoKα) = 0.71073 Å). Diffraction data for a single crystal of compound **2** were obtained at 120 K on an Rigaku Oxford Diffraction SuperNovaII diffractometer equipped with a two-dimensional Atlas detector (rotating anode, λ(CuKα) = 1.54184 Å). Integration, absorption correction, and determination of unit cell parameters were performed using the CrysAlisPro program package [1]. The structures were solved by direct methods and refined by the full-matrix least squares technique in the anisotropic approximation (except hydrogen

atoms) using the SHELX-2013 software [2]. Positions of hydrogen atoms of the organic ligands were calculated geometrically and refined in a riding model. The diffraction data for **2** were weak, and the values of $I / \sigma(I)$ at a resolution higher than 1.2 does not exceed 3 ($R_{int} > 0.20$). However, the structure of the material is sufficiently reliable and clear.

Table S1 contains the crystal data and structure refinement details for **1** and **2**. Selected bond lengths and angles for **1** and **2** are provided in Tables S2 and S3, respectively. The coordination environment of the metal atoms in **1** is shown on Fig. S6. The connectivity of the metal-organic framework **1** is shown on Fig. S7. The coordination environment of zinc(II) cations in **2** is displayed on Fig. S8.

CCDC 1504745 and 1504746 contain the supplementary crystallographic data for this paper. These data can be obtained free of charge from The Cambridge Crystallographic Data Center at http://www.ccdc.cam.ac.uk/data_request/cif.

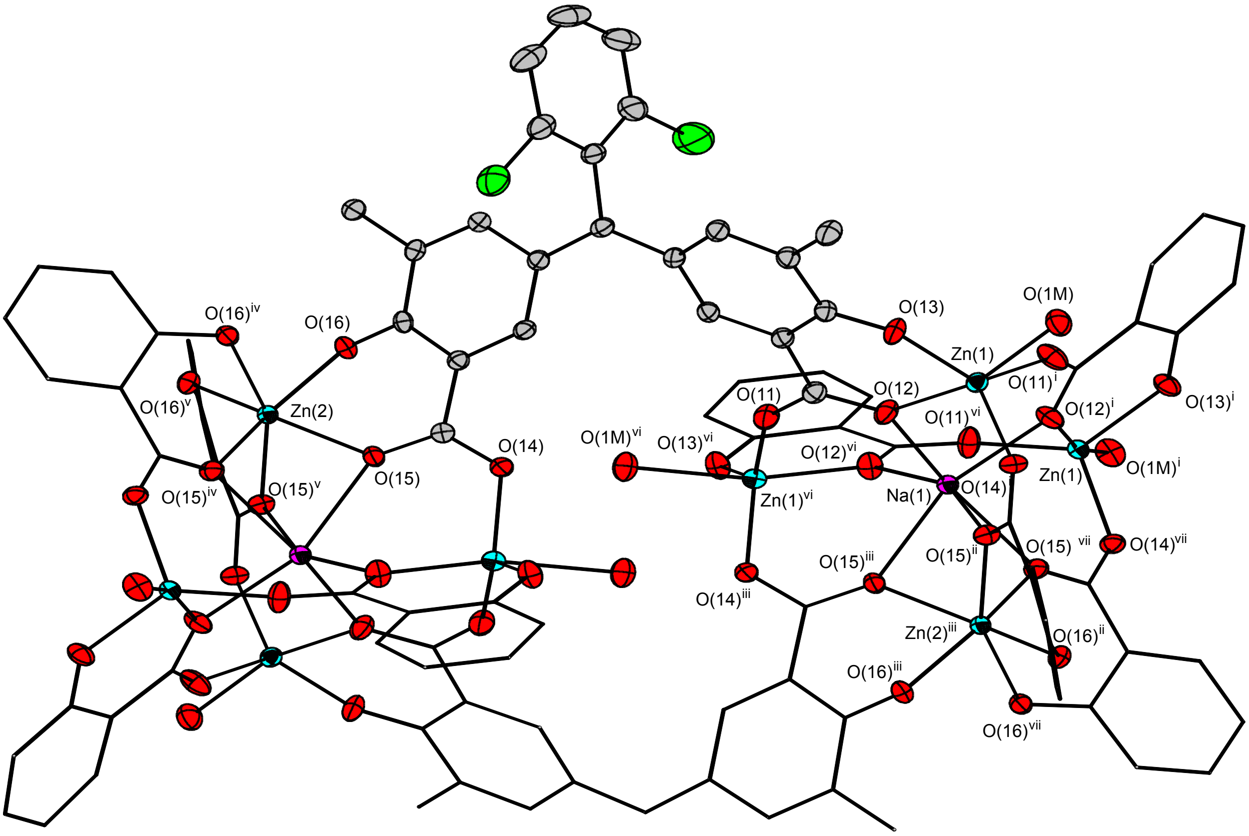

Figure S6. Coordination environment of the metal atoms in **1**. Cryallographically independent atoms and coordination spheres of metal cations are shown in ellipsoids (50% probability). Hydrogen atoms are omitted. Symmetry transformations used to generate equivalent atoms: i) $-x+y+1, -x+1, z$; ii) $x-y+1, x, -z+1$; iii) $-x+1, -y+1, -z+1$; iv) $-y+1, x-y+1, z$; v) $-x+y, -x+1, z$; vi) $-y+1, x-y, z$; vii) $y, -x+y, -z+1$.

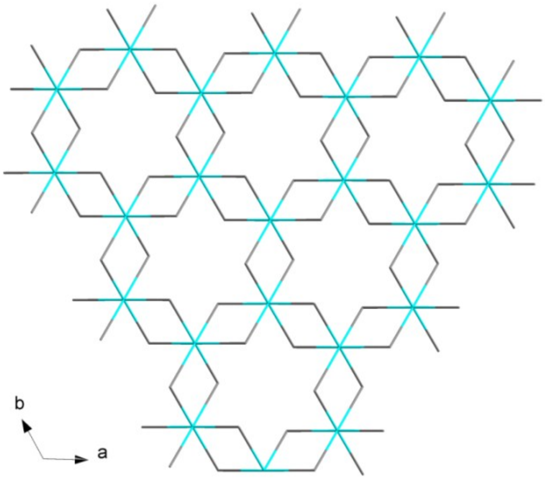

Figure S7. Representation of the layer topology in **1**. Pentanuclear metal clusters are presented in blue; bridging ligands are presented in grey color.

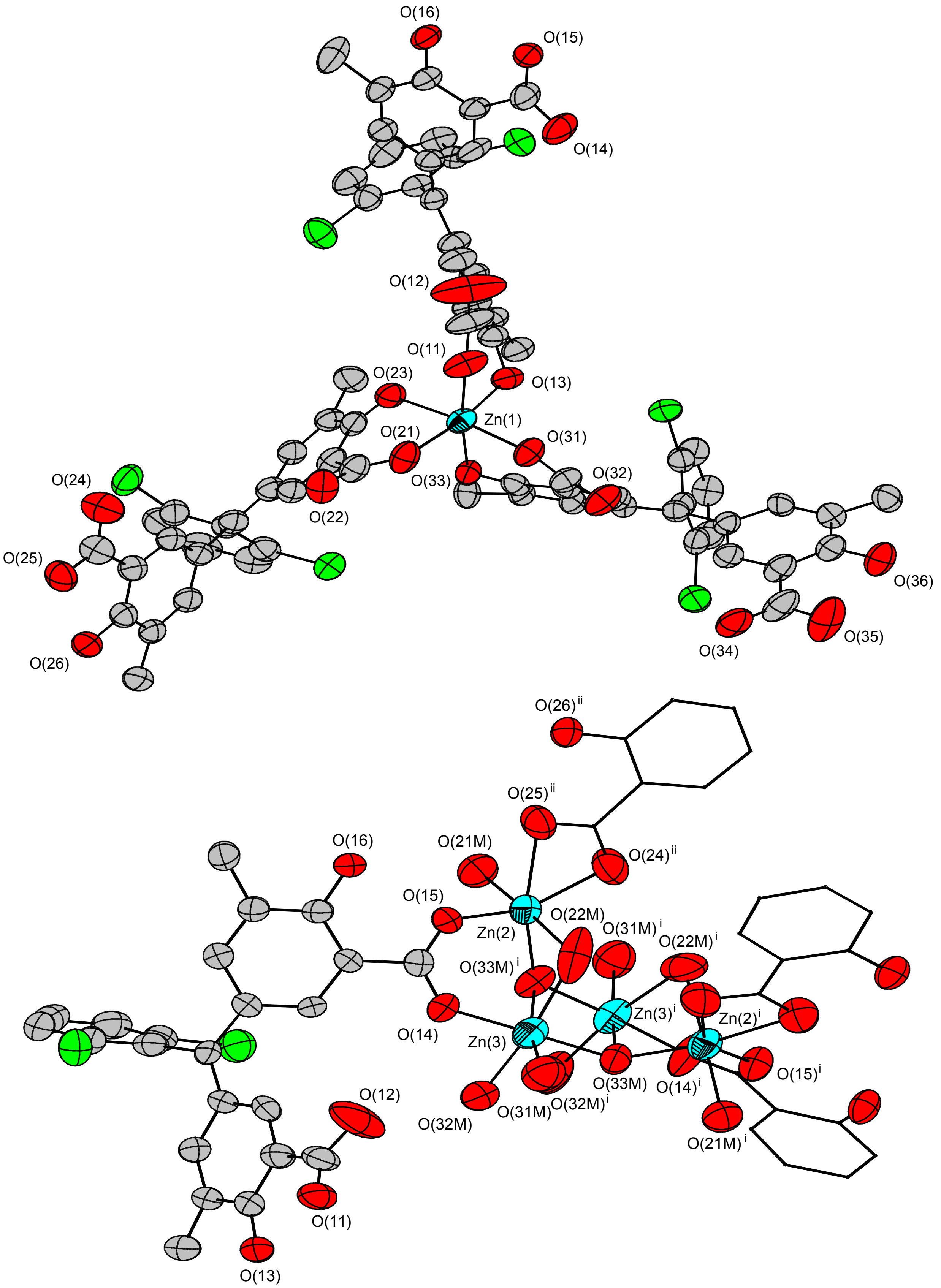

Fig. S8. Coordination environment of Zn(1) (*top*), Zn(2) and Zn(3) (*bottom*) atoms in **2**. Cryallographically independent atoms and coordination spheres of metal cations are shown in ellipsoids (50% probability). Hydrogen atoms are omitted. Symmetry transformations used to generate equivalent atoms: i) $-x + 3/2, y, -z + 1$; ii) $x + ½, -y + ½, -z + 1$.

Table S1. Crystal data and structure refinement for **1** and **2**.

Compound	**1**	**2**
Empirical formula	$C_{81}H_{75}Cl_6NaO_{27}Zn_4$	$C_{73}H_{66}Cl_6O_{27}Zn_3$
Formula weight	1977.58	1784.06
Crystal system	Trigonal	Tetragonal
Space group	$R-3$	$I4_1/acd$
a, Å	19.0345(2)	36.5743(5)
b, Å	19.0345(2)	36.5743(5)
c, Å	41.0567(5)	52.9725(16)
V, Å^3	12882.4(3)	70860(3)
Z	6	32
D(calcd), g/cm^3	1.529	1.338
μ, mm^{-1}	1.373	3.212
F(000)	6060	29184
Crystal size, mm	0.26 × 0.16 × 0.15	0.15 × 0.13 × 0.11
θ range for data collection, deg.	3.31–29.52	2.83–74.59
Index ranges	$-23 \le h \le 25$,<br>$-23 \le k \le 24$,<br>$-56 \le l \le 39$	$-37 \le h \le 42$,<br>$-42 \le k \le 44$,<br>$-64 \le l \le 66$
Reflections collected / independent	16264 / 6829	225414 / 17934
R_{int}	0.0179	0.2612
Reflections with $I > 2\sigma(I)$	5810	12524
Goodness-of-fit on F^2	1.054	1.782
Final R indices [$I > 2\sigma(I)$]	$R_1 = 0.0392$, $wR_2 = 0.1100$	$R_1 = 0.2093$, $wR_2 = 0.4662$
R indices (all data)	$R_1 = 0.0489$, $wR_2 = 0.1169$	$R_1 = 0.2346$, $wR_2 = 0.4890$
Largest diff. peak / hole, e/Å^3	1.413 / −0.760	3.630 / −2.557

Table S2. Selected bond lengths and angles for **1**.

Bond	*d*, Å	Bond	*d*, Å
Zn(1)–O(1M)	2.2216(19)	Zn(2)–O(15)	2.1121(17)
Zn(1)–O(11)^{i}	1.9618(18)	Zn(2)–O(16)	2.0466(17)
Zn(1)–O(12)	2.0794(18)	Na(1)–O(12)	2.2573(19)
Zn(1)–O(13)	1.9680(18)	Na(1)–O(15)^{iii}	2.334(2)
Zn(1)–O(14)^{ii}	1.9908(17)		
**Angle**	**ω, deg.**	**Angle**	**ω, deg.**
O(11)^{i}–Zn(1)–O(1M)	83.14(8)	O(16)–Zn(2)–O(15)	82.70(7)
O(11)^{i}–Zn(1)–O(12)	95.57(8)	O(16)–Zn(2)–O(15)^{v}	112.50(7)
O(11)^{i}–Zn(1)–O(13)	131.98(8)	O(16)–Zn(2)–O(15)^{iv}	157.46(7)
O(11)^{i}–Zn(1)–O(14)^{ii}	111.36(8)	O(16)^{iv}–Zn(2)–O(16)	85.80(7)
O(12)–Zn(1)–O(1M)	163.81(7)	O(12)–Na(1)–O(12)^{vi}	97.19(8)
O(13)–Zn(1)–O(1M)	82.29(7)	O(12)–Na(1)–O(15)^{ii}	83.17(6)
O(13)–Zn(1)–O(12)	86.73(7)	O(12)–Na(1)–O(15)^{iii}	105.88(7)
O(13)–Zn(1)–O(14)^{ii}	113.73(8)	O(12)–Na(1)–O(15)^{vii}	156.73(8)
O(14)^{ii}–Zn(1)–O(1M)	88.60(7)	O(15)^{vii}–Na(1)–O(15)^{iii}	74.44(8)
O(14)^{ii}–Zn(1)–O(12)	106.77(7)	Zn(1)–O(12)–Na(1)	103.54(7)
O(15)^{iv}–Zn(2)–O(15)	83.89(7)	Zn(2)–O(15)–Na(1)^{iii}	85.17(6)
O(15)^{iv}–Zn(2)–O(15)^{v}	83.90(7)		

Symmetry transformations used to generate equivalent atoms:
i) $-x + y + 1, -x + 1, z$; ii) $x - y + 1, x, -z + 1$; iii) $-x + 1, -y + 1, -z + 1$;
iv) $-y + 1, x - y + 1, z$; v) $-x + y, -x + 1, z$; vi) $-y + 1, x - y, z$; vii) $y, -x + y, -z + 1$.

Table S3. Selected bond lengths and angles for **2**.

Bond	*d*, Å	Bond	*d*, Å
Zn(1)–O(11)	2.025(9)	Zn(2)–O(15)	1.992(8)
Zn(1)–O(13)	2.132(8)	Zn(2)–O(24)^{ii}	2.100(11)
Zn(1)–O(21)	2.040(9)	Zn(2)–O(25)^{ii}	2.384(10)
Zn(1)–O(23)	2.128(7)	Zn(3)–O(22M)	2.347(17)
Zn(1)–O(31)	2.018(6)	Zn(3)–O(31M)	2.089(11)
Zn(1)–O(33)	2.116(7)	Zn(3)–O(32M)	2.158(12)
Zn(2)–O(21M)	2.105(9)	Zn(3)–O(33M)	2.039(9)
Zn(2)–O(22M)	2.313(12)	Zn(3)–O(33M)^{i}	2.126(9)
Zn(2)–O(33M)^{i}	2.015(8)	Zn(3)–O(14)	2.027(9)
**Angle**	**ω, deg.**	**Angle**	**ω, deg.**
O(11)–Zn(1)–O(13)	85.5(4)	O(15)–Zn(2)–O(22M)	84.6(4)
O(11)–Zn(1)–O(21)	95.9(4)	O(15)–Zn(2)–O(33M)^{i}	101.0(4)
O(11)–Zn(1)–O(23)	94.4(3)	O(15)–Zn(2)–O(24)^{ii}	146.8(4)
O(11)–Zn(1)–O(33)	169.2(4)	O(15)–Zn(2)–O(25)^{ii}	91.1(3)
O(21)–Zn(1)–O(13)	169.1(3)	O(24)^{ii}–Zn(2)–O(21M)	94.4(5)
O(21)–Zn(1)–O(23)	85.7(3)	O(24)^{ii}–Zn(2)–O(22M)	88.0(5)
O(21)–Zn(1)–O(33)	94.9(3)	O(24)^{ii}–Zn(2)–O(25)^{ii}	57.9(4)
O(23)–Zn(1)–O(13)	83.3(3)	O(31M)–Zn(3)–O(22M)	92.0(5)
O(31)–Zn(1)–O(11)	93.5(3)	O(31M)–Zn(3)–O(32M)	96.3(5)
O(31)–Zn(1)–O(13)	97.0(3)	O(31M)–Zn(3)–O(33M)^{i}	167.0(4)
O(31)–Zn(1)–O(21)	93.8(3)	O(32M)–Zn(3)–O(22M)	170.5(4)
O(31)–Zn(1)–O(23)	172.1(3)	O(33M)^{i}–Zn(3)–O(22M)	76.6(4)
O(31)–Zn(1)–O(33)	85.9(3)	O(33M)–Zn(3)–O(22M)	92.9(4)
O(33)–Zn(1)–O(13)	83.9(3)	O(33M)–Zn(3)–O(31M)	91.2(4)
O(33)–Zn(1)–O(23)	86.3(3)	O(33M)–Zn(3)–O(32M)	91.8(4)
O(21M)–Zn(2)–O(22M)	177.5(5)	O(33M)^{i}–Zn(3)–O(32M)	95.7(4)
O(21M)–Zn(2)–O(25)^{ii}	84.2(4)	O(33M)–Zn(3)–O(33M)^{i}	83.4(3)
O(22M)–Zn(2)–O(25)^{ii}	97.8(5)	O(14)–Zn(3)–O(22M)	89.3(4)
O(33M)^{i}–Zn(2)–O(21M)	98.7(4)	O(14)–Zn(3)–O(31M)	91.5(5)
O(33M)^{i}–Zn(2)–O(22M)	79.5(5)	O(14)–Zn(3)–O(32M)	85.6(4)
O(33M)^{i}–Zn(2)–O(24)^{ii}	109.4(4)	O(14)–Zn(3)–O(33M)^{i}	94.4(4)
O(33M)^{i}–Zn(2)–O(25)^{ii}	167.3(4)	O(14)–Zn(3)–O(33M)	176.4(5)
O(15)–Zn(2)–O(21M)	94.0(4)		

Symmetry transformations used to generate equivalent atoms:
i) $-x + 3/2, y, -z + 1$; ii) $x + ½, -y + ½, -z + 1$.

3. Investigation of Halochromism.

Diffuse reflectance spectra of powders were recorded on a Shimadzu UV-3101 PC spectrophotometer in the range 240 nm to 800 nm at room temperature. Kubelka-Munk (KM) spectra were recalculated from the diffuse reflectance spectra of the samples containing 5% of compound dispersed in the $BaSO_4$ matrix. The reflectance data was converted to Kubelka-Munk units, which are proportional to the sample absorbance, by the following equation:

$$KM = \frac{(1-R)^2}{2R}$$

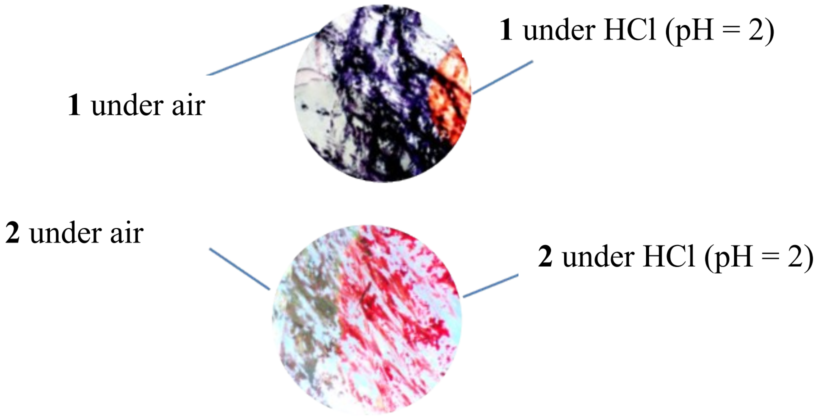

Fig. S9. View of halochromism in solutions for thin layer of **1** (top) and **2** (bottom) under different conditions.

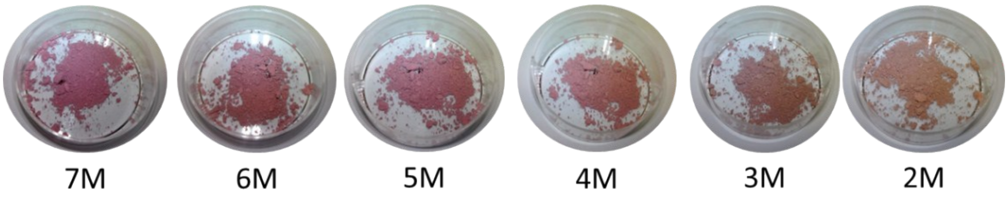

Fig. S10. Photographs of **1**@$BaSO_4$ in vapors of the hydrochloric acid of different concentration.

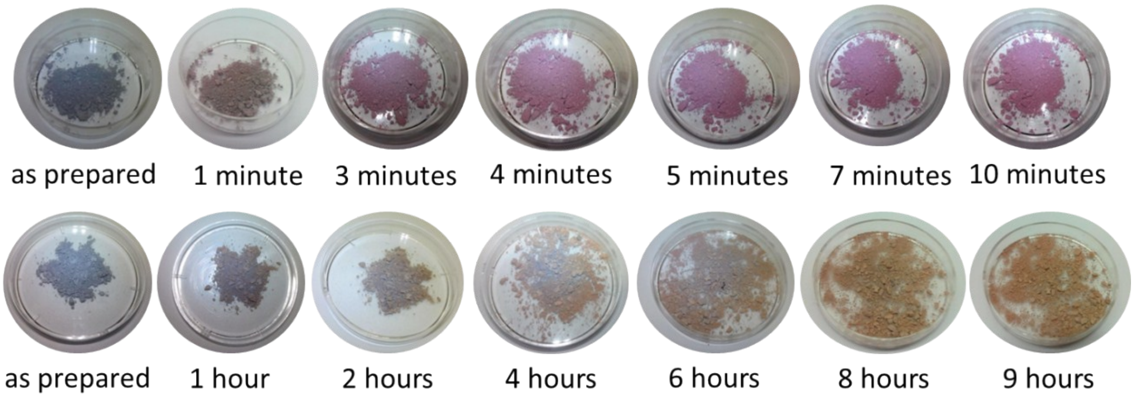

Fig. S11. Photographs of powdered sample of **1**@$BaSO_4$ in vapors of 7M (top) and 2M (bottom) HCl.

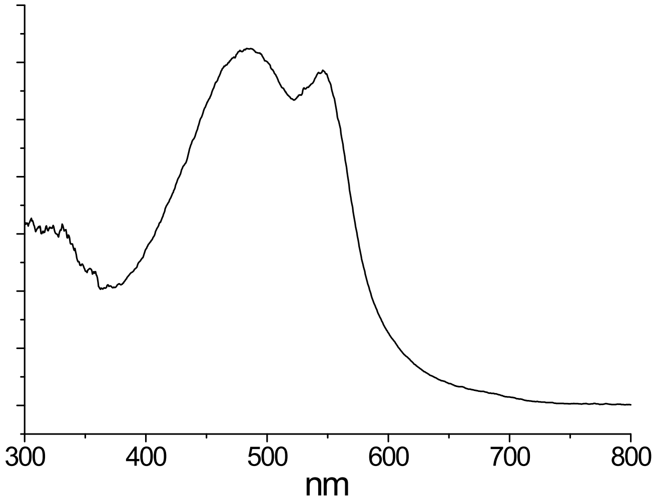

Fig. S12. Diffuse reflectance spectra of **1**@$BaSO_4$ saturated in the vapours of 5M HCl. The spectrum comprises two components at 480 (H_3L form) and 550 nm (H_4L^+ form).

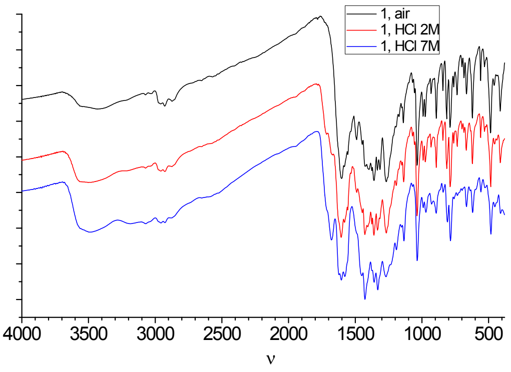

a

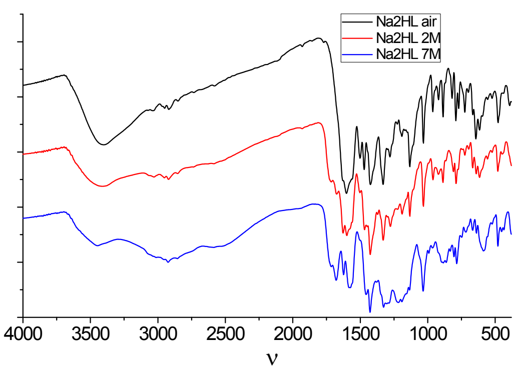

b

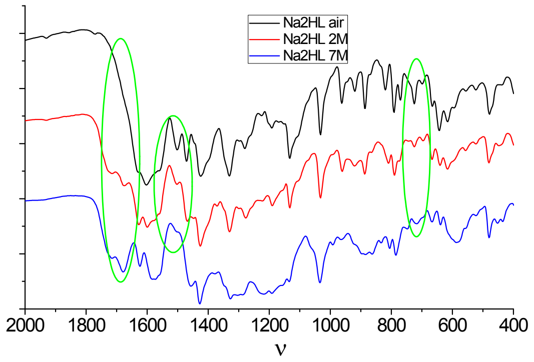

c

Fig. S13. IR spectra for (a) **1** and (b) Chromeazurol B exposed to air (black), under 2M HCl vapors (red), under 7M HCl vapors (blue), (c) Chromeazurol B in the range 400-2000 cm^{-1}.

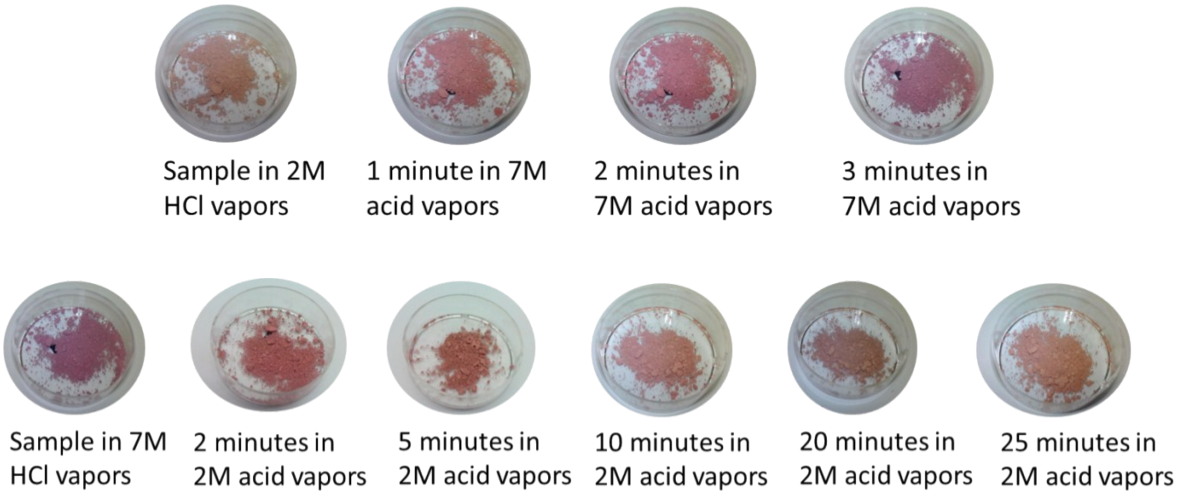

Fig. S14. Gradual changes of color over the time of **1**@B$_{A}$SO$_4$ sample from 2M HCl to 7M HCl (top) and from 7M HCl to 2M HCl (bottom).

pH titration of samples treated under vapors of HCl_{aq}

Measurements were carried out using a Mettler Toledo SevenCompact pH/Ion S220 pH meter at 25°C. Samples of powdered **1** were treated by vapors of 7M and 2M HCl for 1 h. The samples were then exposed to air for several minutes to remove surface-adsorbed HCl. The pH was measured after the addition of distilled water to the resulting solids. The number of protons was calculated by the formula:

$$n = \frac{10^{-pH} \cdot V}{3 \cdot m/1978}$$

where V is the volume of water added, m is the weight of the sample, and 1978 is the molar mass of $[NaZn_4(H_2O)_3(L)_3]\cdot 3THF\cdot 3H_2O$,. As could be seen from the data in Table S1, the number of acidic protons drops after the first minute of exposure to air and stabilizes at 2.8 and 3.9 H^+ per ligand for the number of the protons in **1** after the exposure to vapors 2M and 7M HCl, respectively.

Table S4. pH measurements for samples of **1** exposed to HCl and the experimental determination of the number of protons per ligand in protonated **1**.

For 2M HCl vapors				
weights of polymer sample, mg	time of air exposure, min	volume of water, ml	steady pH	number of protons per ligand (n)
5.5	1	4.00	2.21	3.0
4.7	3	3.00	2.17	2.8
4.8	3	3.00	2.16	2.8
4.7	4	3.00	2.17	2.8
3.2	10	2.00	2.22	2.5
For 7M HCl vapors				
5.2	1	4.00	1.97	5.4
5.0	3	3.00	2.01	3.9
4.7	4	3.00	2.03	3.9
5.4	4	3.00	1.98	3.8
5.0	10	3.00	2.04	3.6

4. References

[1] CrysAlisPro, Agilent Technologies, Version 1.171.37.35 (release 13-08-2014 CrysAlis171.NET).

[2] Sheldrick G.M., *Acta Crystallogr.*, 2008, **A64**, 112-122.
